# A Comparative Study to Analyze the Cost of Curative Care at Primary Health Center in Ahmedabad

**DOI:** 10.4103/0970-0218.62585

**Published:** 2010-01

**Authors:** Neeta Mathur, Geeta Kedia, Atul Trivedi

**Affiliations:** Department of Community Medicine, B. J. Medical College, New Civil Hospital Campus, Asarwa, Ahmedabad-380 016, India

**Keywords:** Capital cost, outdoor patient department, primary health center, reproductive and child health, recurrent cost, subcenter, total cost, unit cost

## Abstract

**Objectives:**

To determine the unit cost of curative care provided at Primary Health Centers (PHCs) and to examine the variation in unit cost in different PHCs.

**Materials and Methods:**

The present study was carried out in three PHCs of Ahmedabad district namely Sanathal, Nandej, and Uperdal, between 1 April, 2006 and 31 March, 2007. For estimating the cost of a health program, information on all the physical and human resources that were basic inputs to the PHC services were collected and grouped into two categories, non-recurrent (capital resources vehicles, buildings, etc.) and recurrent resources (salaries, drugs, vaccines, contraceptives, maintenance, etc.). To generate the required data, two types of schedules were developed, daily time schedule and PHC/SC (Subcenter) information schedule.

**Results:**

The unit cost of curative care was lowest (Rs. 29.43) for the Sanathal PHC and highest (Rs. 88.26) for the Uperdal PHC, followed by the Nandej PHC with Rs. 40.88, implying severe underutilization of curative care at the Uperdal PHC.

**Conclusions:**

Location of health facilities is a problem at many places. As relocation is not possible or even feasible, strengthening of infrastructure and facilities at these centers can be taken up immediately.

## Introduction

Economic issues have had a growing importance in the healthcare field as the sector's share of the gross national product has risen. The health sectors in many countries today are faced with severe resource constraints. Primary healthcare program managers must therefore use the available resources as efficiently and effectively as possible. The optimal use of the resources requires clear and accurate information on the resource flow and on the impact that resources have on the quality and performance of health services.(1)

In India the research on costing of health services is in the nascent stage. Very few studies focus on the detailed analysis of costs and methodological issues in the costing of a service.(2–4) This information is helpful for proper planning and effective management of the limited resources available in the PHCs. Specifically, the purpose of this study is to analyze the costs of government hospitals (Primary Health Center), at the village level, in providing curative services and to examine the variation in unit cost in different PHCs.

## Materials and Methods

### Sample design

There were 46 PHCs in the Ahmedabad district. All the PHCs were stratified into two groups, based on the performance of their RCH indicators [Table 1] in the year 2005 – 2006 (one group with good performance and the other group average). There were 17 good performing PHCs and 29 average performing PHCs. From these two categories, three PHCs were randomly selected one from the good performing PHCs (Sanathal) and two from the average performing PHCs (Nandej and Uperdal). Information on the cost of equipment, building, staff salary, and so on, was collected from the selected PHCs and all the subcenters under the jurisdiction of these selected PHCs.

**Table 1:** List of 13 RCH indicators used to categorize PHCs of Ahmedabad district

S. No.	RCH Indicators
1	Total ANC registration
2	Early ANC registration
3	ANC 3 check-up
4	Total delivery registration
5	Institutional delivery
6	PNC 3 check-up
7	TT mother
8	BCG
9	DPT 3/Polio 3
10	Measles
11	Fully immunization
12	Sterilization
13	IUD

### Background information of primary health centers

Sanathal PHC is located on main state highway and is well connected to the community health center (CHC) as well as the subcenters, Nandej PHC is located on the railway route, whereas, the Uperdal PHC is far away from the nearest CHC, and also roadway conveyance is poorly available and the subcenter connectivity is poor.

It can be seen that the selected Primary Health Centers cover more population than the prescribed limit of population (30,000 for Rural PHC). The number of subcenters exists according to the prescribed norms [Table 2]. The organizational structure of the PHCs selected for the study broadly conform to the pattern recommended for such institutions by the Government of India and the Government of Gujarat.

**Table 2:** Background information about selected PHCs and population covered for the study

Name of taluka	Sanand	Daskroi	Sanand
Type of population	Rural	Rural	Rural
Population of taluka	2,15,836	2,72,000	2,15,836
Name of FRU (CHC) for the	CHC-Sanad	CHC-	CHC-Sanad
above taluka		Singerva	
Name of selected PHC	Sanathal	Nandej	Uperdal
No. of subcenter under PHC	6	6	7
Population covered by PHC	36,846	34,171	41,290
No. of eligible couples	6,087	5,537	7,080

The staff pattern shows that at the Sanathal PHC, two posts for Multi Purpose Workers (MPW) — Male, are vacant and two posts for Multi Purpose Workers (MPW) — Female, are vacant, whereas, in the Nandej PHC, two posts for MPW — Male, are vacant, and at the Uperdal PHC one post for MPW — Female, is vacant [Table 3]. Laboratory Technician facility is available at all the three PHCs, while Driver is posted only in Nandej PHC. Wherever posts for paramedical staff are vacant, the staff in position has to work extra. This study has focused on only internal issues and not collected data regarding the availability, accessibility, and efficiency of the nearby private medical care.

**Table 3:** Staff pattern at the selected primary health center as on April 2006

Staff/Category	(NIH-FIC)		Nandej		Uperdal	
			
	Sanctioned	In position	Sanctioned	In position	Sanctioned	In position
Medical officer	1	1	1	1	1	1
Pharmacist	1	1	1	1	1	1
Lab technician	1	1	1	1	1	1
PHN/FHS	1	1	1	1	1	1
HA/MHS	1	1	1	1	1	1
MPW – Female	6	4	6	6	7	6
MPW – Male	4	2	4	2	4	4
Driver	-	-	1	1	-	-
Class IV	1	1	2	2	1	1

**Table 4:** List of items for costing in the PHC and their sources of data as on April 2006

List of items	Source of data
Capital cost	
Building	RCH office, District Health Office, Ahmedabad(5)
Vehicle	Wholesale dealer of vehicles
Equipments	CMSO, Gandhinagar
Furniture	RCH office, District Health Office, Ahmedabad
Electrical installations	RCH office, District Health Office, Ahmedabad
Recurrent cost	
Salaries of personnel	Records of salary, BHO
Drugs and consumables	CMSO, Gandhinagar(6)
Electricity bills	Records of electricity bills, BHO
Diesel bills	Records of diesel bills, BHO
Telephone bills	Records of telephone bills, BHO
Building maintenance	Receipt of maintenance, RCH office, District Health Office, Ahmedabad

**Table 5:** Allocation statistics used for various capital and recurrent costs

Inputs	Statistics used for allocation
Recurrent cost	
Salaries of personnel	Total time spent by the employees in each concerned service
Consumables	Based on indents consumed
Electricity charges	Total cost of electrical appliances and electrical gadget fittings in each service unit
Telephone charges	Time used in the concerned service
Building maintenance	Time used in the service
Vehicle charges	Allocated equally to the concerned service
Capital cost	
Building	Time used by the service
Furniture	Total cost of furniture in each service unit
Electrical installation	Total cost of electrical appliances and electrical gadget fittings in each service unit
Appliance and equipment	Total cost of appliances and equipment in each service unit
Vehicle	Allocated equally to the concerned service

**Table 6:** Cost allocation for curative care in different PHCs during the year 2006–2007

Items of expenditure	Sanathal	Nandej	Uperdal
Recurrent cost			
Salary	222825.20	238169.90	316908.93
Consumables	137264.80	208600.20	274859.50
Electrical charges	1658.37	1733.32	1146.77
Telephone charges	0.00	5031.18	0.00
Building maintenance charges	2554.81	2554.81	2554.81
Vehicle charges	0.00	0.00	0.00
Total recurrent cost (Rs.)	3,64,303.17	4,56,089.41	5,95,470.02
Capital cost			
Phc building depreciation	8516.00	8516.00	8516.00
Subcenter building depreciation (6)	4193.68	4193.68	4892.62
Furniture depreciation	4177.18	3718.77	2267.24
Electrical fitting depreciation	306.81	289.24	296.47
Appliance depreciation	4170.78	3963.35	979.19
Vehicle depreciation	0.00	0.00	0.00
Total capital cost (Rs.)	21,364.44	20,681.02	16951.52
Total cost (Rs.)	3,85,667.61	4,76,770.43	6,12,421.54
Cases handled	13103	11664	6939
Unit cost (Rs.)	29.43	40.88	88.26

**Table 7:** Family planning services performance in different PHCs during the year 2006–2007 (in percentage)

Indicator	Sanathal	Nandej	Uperdal
Sterilization	100	91	100
IUD	100	94	100
Oral pills users	128	107	103
Condom users	99	83	125
Total	101	88	116

**Table 8:** MCH program performance in different PHCs during the year 2006–2007 (in percentage)

Indicator	Sanathal	Nandej	Uperdal
Total ANC registered	105	98	106
Early ANC registered	58	45	57
ANC 3 checkup received	91	51	69
Total delivery registered	103	95	97
Institutional delivery	83	57	28
PNC 3 checkup received	80	81	74
Total	87	71	72

**Table 9:** Immunization services performance in different PHCs during the year 2006–2007 (in percentage)

Indicator	Sanathal	Nandej	Uperdal
TT mother	102	102	101
DPT 3	105	98	103
Polio 3	105	98	100
BCG	114	99	106
Measles	105	101	102
Fully immunized	104	101	102
Total	106	100	102

### Time period

Financial year 2006–2007 (1 April, 2006 to 31 March, 2007) was taken as the study period. This is probably consistent with the records of most types of relevant data, such as, expenditure on personnel and services provided. A one-year period avoids any distortions that might be caused by seasonal effects.

### Data collection

This study utilized a variety of methods for collecting data from the district, Block Health Office (BHO), PHC, and Subcenter level; depending upon the nature, type, quality, and quantity of data requirements as per the objectives of the study. The list of items for costing with the source of information on each of them is given in Table 4.

### Allocation of cost

Costs of various resources were allocated into various programs according to their uses in the concerned programs. Table 5 shows the allocation statistics used for various inputs.

- Resources that were being used exclusively to produce only one type of function or service such as curative care or MCH or family planning or any other program such as malaria.
- Resources that were being used to produce more than one type of function or service. For example, health functionaries being multipurpose workers, their services were utilized for all programs.
- Resources that did not produce any function or service, but were used to support general operations; for example, sweeper or room used for storage, waiting space, and so on.

Allocation of cost for the first group of resources was allotted against the concerned programs. Therefore, if a building or equipment was used especially for an MCH program, the annualized capital cost of the building or equipment was allotted against the MCH program.

Allocation of cost for the second group of resources was done on the basis of the per cent of time spent by the workers on that activity. The cost of such resources was allocated to the appropriate program categories in the same proportion as the Direct Service Time of those programs.

For the third group of resources, that is, the resources that were being used only as a support service, the cost allocation for the Service Programs was done equally.

Utilization of Time Use Data for Cost Estimation: The allocation of the total cost for different programs was done on the basis of the proportion of time spent by different health functionaries on various programs. For this, a specially developed time use form was provided to the doctors, supervisors, and workers, for reporting their daily activities. These schedules were filled up every day for six consecutive working days. To discourage filling the forms at the end of the day or at a later date, it was instructed to fill up the schedule after finishing some activity. Thus each worker reported activities carried out for direct services (curative care, FP, MCH, and other programs), support services (supervision, waiting time, traveling time, record keeping administration, etc.).

For estimating the time devoted to different activities, the units attained for different activities were summed. Before summing up, some initial checking of the information on every unit was done. If, for the same 30 minute period both direct services (resulting in an immediate output) and support services (facilitating production of different services) were reported, only direct services were considered. However, if more than one direct service was performed, one unit was divided equally among as many direct services as were provided during a period of 30 minutes. Units for support service activities were also summed up in a similar manner.

The following definitions were used to calculate the costs

#### Cost

The value of resources used to produce something, including a specific health service or a set of services

#### Total cost

For estimating the cost of the health program, all inputs were classified into two groups, non-recurrent (capital) resources and recurrent resources, those that are used up in the course of a year and usually purchased regularly (i.e., recurrent costs) and those that last longer than one year, such as buildings, vehicles, and equipments (i.e., capital cost). Total cost is the sum of recurrent and capital costs.

#### Unit cost

Unit cost is a simple average or the cost per unit of outcome (i.e., an indicator of efficiency). The basic calculation of a unit cost is average cost per total number of beneficiaries who were provided OPD and Indoor services at the PHC/SC.

### Limitations

In the process, a number of assumptions and limitations had to be framed in the study.

Actual monitoring of the Health Staff activities was not possible in the field. Therefore, the time spread sheet filled by them was considered as it was.

Time spent on traveling and unproductive activities was not calculated as it was not possible to cross check.

## Results and Discussion

In the present study, we compared the unit costs of curative care at the selected PHCs (Sanathal, Nandej, and Uperdal). The comparison indicated that the Sanathal PHC, with good performance, had a low unit cost in curative care compared to the Nandej and Uperdal PHCs, where a high unit cost was seen in curative care. Various operational performance indicators of the Sanathal PHC, Nandej PHC, and Uperdal PHC are provided in Table 6; using this information the unit cost of curative care has been worked out.

The total cost incurred in curative care is Rs. 3.85 lakhs at the Sanathal PHC, Rs. 4.76 lakhs at the Nandej PHC, and Rs. 6.12 lakhs at the Uperdal PHC. The unit cost of curative care is the lowest (Rs. 29.43) for the Sanathal PHC and highest (Rs. 88.26) for the Uperdal PHC, followed by the Nandej PHC with Rs. 40.88. The unit cost on curative care is highest for the Uperdal PHC; this may be because of two reasons; first, the inflow of patients is in the range of 15 to 25 patients per day compared to the inflow of patients in the range of 30 to 40 patients per day at the Sanathal and Nandej PHCs, and second, accessibility of the PHC is poor. Similarly, Bhat R,(7) in a study, reported that the cost of handling OPD patients, per case, works out to be Rs. 57 in the Sanand CHC, which is well equipped in terms of physical infrastructure.

**Figure 1:**
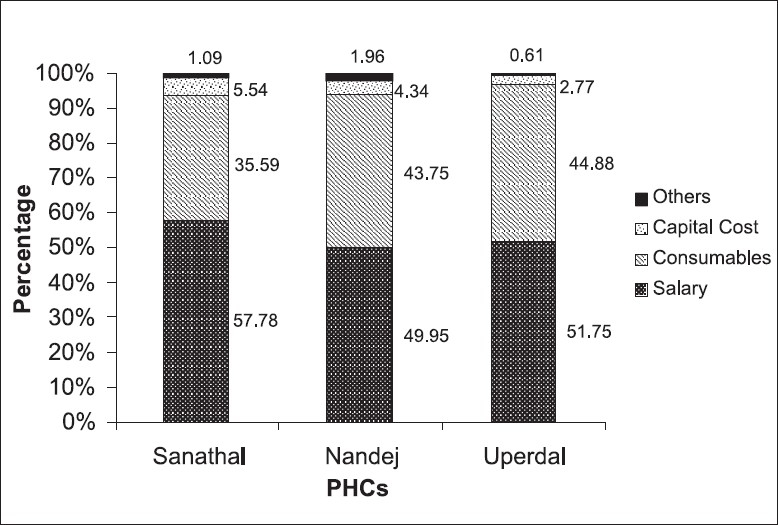
Classification of total cost by components for curative care in different PHCs during the year 2006–2007

Figure 1 shows the percentage of the recurrent and capital costs in the total costs of curative care. It is observed that the percentage of capital cost is lowest for the Uperdal PHC (2.77 %), whereas, for the Sanathal PHC it is relatively higher (5.54%), mainly because of the high appliance (19.52 %) and furniture cost (19.55%). The Sanathal PHC OPD is well equipped with computer facility, along with other furniture such as a soft board for display of articles. For the Nandej PHC, the percentage of capital cost is 4.34%.

On further decomposition of capital and recurrent costs it is observed that the personnel cost is the main category among the recurrent costs accounting for about 53 – 61% of the total recurrent cost. It is followed by the expenses on drugs. Although the composition is broadly similar in all PHCs, there are some minor variations. Uperdal PHC has the largest percentage of cost incurred on drugs (46.16%), while Sanathal PHC comes up with the smallest percentage (37.68%) on drugs. The high unit cost of the Uperdal PHC could be caused on account of the large share of drugs. Similar findings are seen in the study conducted by Chitre VS,(8) on the public hospitals in Maharashtra where the high unit cost of OPD was on account of drugs (20.23%) in the Thane hospital.

### Per capita expenditure

The Per Capita Expenditure (PCE) on a service is defined as the total expenditure incurred for the service per person. For example

PCE on curative care = Total expenditure on curative care in the in an accounting year = accounting year in a PHC = Total population in the accounting year

Per capita expenditure for curative care is lowest at the Sanathal PHC Rs. 10.47 followed by Rs. 13.95 at the Nandej PHC, and Rs. 14.83 at the Uperdal PHC.

### Performance indicator in various programs

The performance indicator in various programs is calculated by dividing the output measures of a particular indicator from its workload. Table 7 presents the performance indicator of the Family Planning services. Overall 100% of the Family Planning services are achieved by the Sanathal and Uperdal PHCs, whereas, the Nandej PHC has achieved the target only in oral pill users. The performance of condom use is 125% in the Uperdal PHC, whereas, 83% in the Nandej PHC.

Table 8 presents the performance indicator of the MCH program. The overall performance is good in the Sanathal PHC (87%) compared to the Nandej (71%) and Uperdal PHCs (72%). Institutional delivery is lowest in the Uperdal PHC (28%). Early ANC registration is only about half of the total ANC registered in all the PHCs. PNC 3 check up received is around 80%. The Sanathal PHC has achieved its target in a total ANC registered and total delivery registered.

Table 9 presents a performance indicator of Immunization services. All the three PHCs have achieved the target of 100% immunization with the Sanathal PHC 106%, Nandej PHC 100%, and Uperdal PHC 102% achievement. Only Nandej PHC lags behind in the following indicators DPT 3 (98%), Polio 3 (98%), and BCG coverage (99%).

## Conclusion

The unit cost of curative care is the lowest (Rs. 29.43) for the Sanathal PHC and highest (Rs. 88.26) for the Uperdal PHC, followed by the Nandej PHC with Rs. 40.88, implying severe underutilization of OPDs at the Uperdal PHC. Component specific expenditure shows that a majority of the total expenditure was accounted for by expenditure on the staff. The per capita expenditure on Curative care was lowest at the Sanathal PHC followed by the Nandej PHC and Uperdal PHC.

### Recommendations

On the basis of this study the following recommendations are made for better utilization of health care services at the Primary Health Center. (i) Location of health facilities is a problem at many places. As relocation is not possible or even feasible, strengthening of infrastructure and facilities at these centers can be taken up immediately. (ii) Increasing the coverage of health services is a key to reduce the unit cost. (iii) Personnel costs account for the maximum share (about two-thirds) of the total cost. Hence, efforts should be made to have a judicious use of personnel.
